# E-Health Interventions to Improve Health Outcomes in Patients with Systemic Lupus Erythematosus: A Systematic Review

**DOI:** 10.3390/healthcare12161603

**Published:** 2024-08-12

**Authors:** Ana Canal-Pérez, Alba Navas-Otero, Araceli Ortiz-Rubio, Alejandro Heredia-Ciuró, Julia Raya-Benítez, Javier Martín-Núñez, Marie Carmen Valenza

**Affiliations:** 1 Department of Physiotherapy, Faculty of Health Sciences, University of Granada, Av. de la Ilustración 60, 18016 Granada, Spain; anacanal@correo.ugr.es (A.C.-P.); ahc@ugr.es (A.H.-C.); javimn@ugr.es (J.M.-N.); cvalenza@ugr.es (M.C.V.); 2 Department of Nursing, Faculty of Health Sciences, University of Granada, Av. de la Ilustración 60, 18016 Granada, Spain; juliarb@ugr.es

**Keywords:** autoimmune disease, disease management, telemedicine, health

## Abstract

Background: Systemic lupus erythematosus (SLE) is a heterogeneous autoimmune disease that involves damage to one or more organs and systems. E-Health technologies have been used to improve the quality of care and to minimize the cost of rehabilitation services. This study aimed to provide the most recent and convincing evidence on the rehabilitation effects of e-Health interventions compared to conventional treatments. Methods: A systematic review was conducted. Inclusion criteria were defined following PICO recommendations (i.e., populations, intervention, comparison and outcome measures). Methodological quality and risk-of-bias were assessed for each study. Results: Six studies met the inclusion criteria, providing data on 743 individuals with SLE. Results indicated that e-Health interventions improved health outcomes, such as disease management or emotional status. Methodological quality was moderate and low risk-of-bias was found in the majority of the studies included. Conclusions: For patients with SLE, e-Health interventions are a safe rehabilitation intervention to improve health outcomes. However, more high-quality studies with large samples are needed, with a focus on the long-term outcomes of e-Health interventions for patients with SLE.

## 1. Introduction

Systemic lupus erythematosus (SLE) is a heterogeneous autoimmune disease that involves damage to one or more organs and systems, such as the cardiovascular, pulmonary, and central nervous systems, the kidneys and the skin, and affects the quality of life of patients. The condition is more prevalent in women between 15 and 40 years old. There are several issues that need to be addressed concerning diagnosis and management [1]. Although treatment strategies for SLE have significantly improved, resulting in better prognosis [2], there are still many challenges and unmet needs in the diagnosis and therapeutic management of the disease [2]. For this reason, diagnosis and treatment can be variable and difficult to predict, causing frustration for clinicians and patients and leading to high annual costs [3,4]. 

The main symptoms associated with SLE include physical and mental fatigue, muscle weakness, cognitive impairment, skin rashes, including the malar “butterfly rash”, and anxiety, causing disability [5]. The British Society for Rheumatology guidelines for the management of SLE [6] recommend a combination of pharmacological and non-pharmacological treatment administered by a multidisciplinary team. Despite improvements in care, patients report difficulties in following their treatment plans, resulting in failure to manage their illness. This can adversely affect their health outcomes, including disease management, disability, and quality of life [7,8].

New technologies to support health (i.e., e-Health technologies) have been designed to improve quality of care and minimize costs [9]. In this regard, the recent development and growth of e-Health, defined as “the use of information and communications technology in support of health and health-related fields” [10], stood out during the COVID-19 pandemic in various healthcare settings due to the benefits provided by these types of tools in the quality, efficiency, and accessibility of care. The importance that e-Health tools have acquired in health care has involved a technical, economic, and training investment and such tools are now commonly used by health professionals [10,11,12,13]. In addition to directly helping SLE patients with the management of their disease, e-Health technology provides information that can guide clinical practice, the development of scientific projects, remote monitoring, etc. [9,13]. As a result, e-Health apps are useful for SLE patients, clinicians, and clinical investigators [12].

It is essential to take into account that there are previous systematic reviews and relevant studies exploring similar therapeutic approaches and outcomes [14,15]. Despite the availability of numerous studies that show the effectiveness and benefits of e-Health, its adoption has been relatively slow [9]. Only few studies have specifically focused on e-Health therapeutic approaches. For instance, previous studies have predominantly concentrated on pharmacological treatments, lifestyle modifications, and conventional therapeutic approaches, leaving a clear gap in the exploration of e-Health interventions. A notable systematic review on this topic was published by Debon et al. [14], who explored several e-Health interventions for chronic diseases, including SLE. However, this study [14] does not provide a comprehensive analysis of SLE. Additionally, other reviews [15,16] mainly address the broader spectrum of rheumatologic conditions and do not focus on the specific challenges of SLE. 

To the best of our knowledge, so far no systematic reviews have specifically focused on evaluating the evidence of e-Health interventions in improving health outcomes in patients with SLE. This study aimed to provide the most recent and convincing evidence on the rehabilitation effects of e-Health interventions compared to conventional treatments for SLE patients.

## 2. Materials and Methods

### 2.1. Registration and Protocol

The present study followed the PRISMA (Preferred Reporting Items for Systematic Reviews and Meta-Analyses) considerations [17] and was registered in the International Prospective Register of Systematic Reviews database (PROSPERO: CRD42023473402).

### 2.2. Eligibility Criteria

Eligibility criteria were developed using the PICO [Patients, Interventions, Comparison, Outcome] framework [18]. To be included, studies had to (1) include participants with a clinical diagnosis of SLE; (2) include an arm with an e-Health intervention; and (3) include the assessments of health outcomes. For the study design, we included randomized controlled trials (RCTs). For comparison, we considered any other intervention or treatment as usual. Studies were excluded if they were protocol studies, observational studies, systematic reviews, meta-analyses or studies involving other pathologies.

### 2.3. Information Sources and Search Strategy

Searches were conducted in November 2023 by three members of the study team across the following electronic databases (Medline via Pubmed, Web of Science, Journal Citation Reports, Biosis Citation Index, Biosis Previews, Current Content Connect, Derwent Innovations Index, Korean Journal Database, Preprint Citation Index, SciELO Citation Index). No limitations were established regarding publication date or language. For the search strategies used, the descriptors were defined using Medical Subject Headings (MeSH). The search strategy is described in Supplementary Materials S1.

### 2.4. Selection and Data Collection Process

Data were extracted and verified by three authors using a standardized spreadsheet and following the PICO framework [18]. If additional data were required, study authors were contacted and the authors were given a period of one month to respond (if no response was received, an additional email was sent two weeks after the first email to reinforce the request). The following data were collected from each selected study: (1) author, year and country (data); (2) study design, (3) participant details (e.g., number, mean age, age range, gender); (4) intervention characteristics (e.g., description, comparison, duration, and follow-up period); (5) outcome data; and (6) key findings.

### 2.5. Study Risk-of-Bias Assessment

The study team used the PEDro Scale [19] to conduct the methodological quality assessment. This tool is only applied in experimental studies and consists of a checklist of 11 criteria, of which 10 are scorable. Each criterion that the study meets is awarded 1 point, obtaining a total score of 10 points. For this review, studies with PEDro scores ranging from 6 to 10 were considered as “high quality”, those with scores from 4 to 5 were considered as “moderate quality”, and those with scores from 0 to 3 were considered as “low quality”. The PEDro scale does not evaluate clinical utility.

Moreover, all three authors assessed study quality using Version 2 of the Cochrane risk-of-bias tool for randomized trials (RoB 2) [20]. This tool assesses five domains (i.e., randomization process, deviations from the intended interventions, missing outcomes, measurement of the outcome, and selection of reported results) and provides an overall bias analysis. All studies included in this review were analyzed with this tool. Studies were assessed as “high risk”, “low risk”, or “some concerns”.

## 3. Results

### 3.1. Study Selection

**Figure 1:**
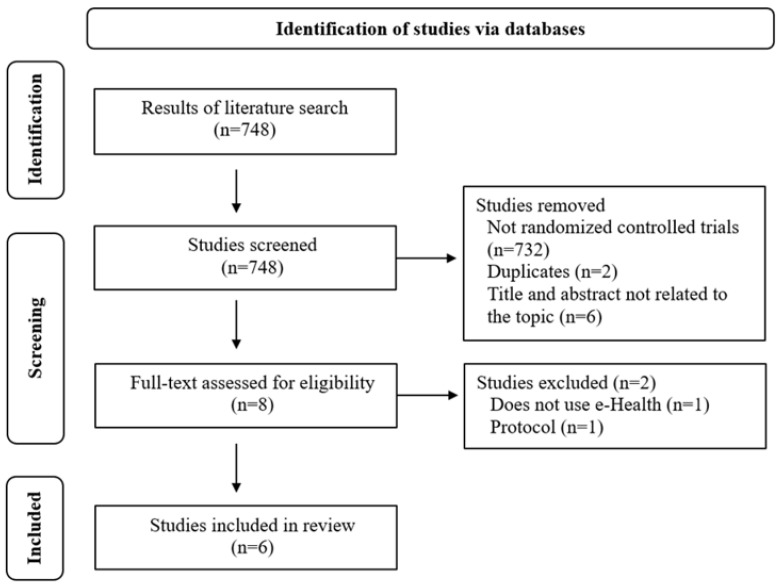
PRISMA flow diagram of literature search and study selection.

In total, 748 results were retrieved through database searches. After applying the different criteria established in the methodology, a total of eight articles were screened. Of these, 8 were selected for full-text review. A total of 6 RCTs were included in the final review. The PRISMA flow diagram of the literature search and study selection is shown in Figure 1.

### 3.2. Study Characteristics

This review included a total of six RCTs [21,22,23,24,25,26]. The total sample size of all studies was 743 participants, all diagnosed with SLE. The age ranged from 20 to 44 years old. Two studies did not report participants’ age [25,26]. All the studies identified the sex of participants, with a predominance of women. Four studies were conducted in the United States [22,24,25,26] and the other two were performed in China [21] and Iran [23]. 

The structure of e-Health interventions was heterogeneous between studies. The studies used different modalities of interventions, such as disease management or supportive counseling, to improve the quality of life of patients. All studies described the comparison groups, but different types of control interventions were described, such as usual care at in-person clinics or pamphlets. The characteristics of the studies included are presented in Table 1. 

The methodological quality of each study was evaluated with the PEDro scale [19], which is summarized in Table 2 for all the identified trials.

Most studies were assessed as “high quality”. Only one was considered as “moderate quality” [23]. There was no concealment or blinding of participants and evaluators in any study.

**Figure 2:**
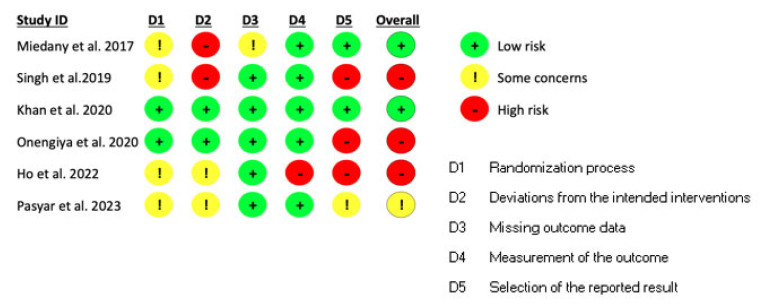
Risk-of-bias of randomized controlled trials included in the systematic review using the RoB 2 [21,22,23,24,25,26].

The quality of each study was evaluated according to Version 2 of the Cochrane risk-of-bias tool for randomized trials (RoB 2) [20], which is summarized in Figure 2 for all the identified trials.

Most studies had not concealed the allocation of participants during the interventions nor had they applied any blinding techniques. Most evaluators were previously aware of participants’ assigned intervention during the trial.

### 3.3. Intervention Characteristics

Of the six articles reviewed, four evaluated the effectiveness of e-Health interventions to improve disease management [21,23,25,26]. Another examined the feasibility of the use of an automated digital reminder to improve medication adherence [24], and one tested the efficacy of the individualized decision aid [22]. The duration of the interventions ranged from 1 to 24 months (median: 6 months). Only one study had information about the frequency of sessions [26]. In that study, e-Health coaching sessions were conducted weekly for 20-30 min over 15 weeks. Only in one study, participants were contacted by phone and email to supervise the intervention [22]. The rest of the studies did not report on the supervision of the intervention [21,23,24,25,26].

### 3.4. Results of Individual Studies

It was not possible to perform a meta-analysis to estimate the overall effect because of insufficient data and heterogeneity between the studies included. However, the main results of this systematic review are presented below.

The study by Ho et al. [21] evaluated short-term patient satisfaction, disease control, and infection risk of telemedicine (i.e., experimental group) in comparison to standard in-person follow-up (i.e., control group) during the COVID-19 pandemic. The mean overall patient satisfaction score was higher in the TM group (2.2 ± 0.6 vs. 1.9 ± 0.8, *p* = 0.042). The proportion of patients who continued on LLDAS was similar between both groups (TM: 75.0% vs. SF: 74.2%, *p* = 0.919). There was no difference between both groups in the SLEDAI-2k (TM: 3.6 ± 1.9 vs. SF: 3.5 ± 2.5, *p* = 0.655), although the PGA was even higher in the TM group (0.52 ± 0.49 vs. 0.36 ± 0.40, *p* = 0.025).

Miedany et al. [25] analyzed the results obtained from monitoring using electronic assessments of patient-reported outcome measures. Monitoring of the active group was carried out by a rheumatology nurse every month. In addition, follow-up was conducted by a rheumatologist every three months. In comparison, the control group had standard management. At the end of 12 months, no discernible differences in systemic SLEDAI or ACR SDI scores were found in either group. However, the control group reported significantly higher SLEDAI scores (*p* < 0.01) than the active group at the end of the study (active group (3.1–2.6) vs. control group (7.6–6.7).

The study of Onengiya [24] tested the efficacy of the SimpleMed+ pillbox, which is an automated digital reminder aimed at improving medication adherence. Results showed that 88% of patients in the treatment group actively used the SimpleMed+ pillbox, reporting no issues with reminders. Baseline objective adherence differed between the control and treatment groups. The control group’s adherence declined significantly over the study, while the treatment group’s adherence continued to increase; the increase was not statistically significant (63 to 66, *p* = n.s.).

The findings of the study by Pasyar et al. [23] revealed that no statistically significant differences between the group that received smartphone support (i.e., experimental group) and the treatment-as-usual group (i.e., control group) in mean health anxiety (HA) scores prior to the intervention (*p* = 0.76). However, after the 2-month intervention, mean HA was significantly lower in the experimental group than in the control group (*p* < 0.001). Before the intervention, there was no significant difference between both groups (*p* = 0.70).

Singh et al. [22] tested the efficacy of the IDEA-WON (Individualized Decision Aid for Diverse Women with Lupus Nephritis) compared to a lupus pamphlet for 3 months. The statistically significant reduction in all DCS subscale scores (*p* < 0.05) was associated with the use of decision aids except for one score: the feeling of support in the decision-making subscale (*p* = 0.056).

Khan et al. [26] sought to showcase how integrating a digital therapeutic intervention featuring telehealth coaching into standard care enhances the quality of life of SLE patients over a 16-week period. To ascertain this, they used the Functional Assessment of Chronic Illness Therapy-Fatigue (FACIT-F), the Brief Pain Inventory-Short Form (BPI-SF), and the Lupus Quality of Life (LupusQoL) questionnaire. The outcomes revealed statistically significant findings favoring the intervention group (i.e., e-Health group) in all tools used.

According to our results, only two studies showed a significant improvement between groups in favor of the e-Health intervention group [21,26]. Among these, the best results were shown by the study by Khan et al. [26], with an intervention based on a mobile app. Participants who received the digital therapeutic intervention featuring telehealth coaching reported the best results in FACIT-F (*p* < 0.001), in BPI-SF in the domain of pain (*p* = 0.02) and in the LupusQoL in the domains of planning (*p* = 0.004), emotional health (*p* = 0.02), and fatigue (*p* < 0.001).

## 4. Discussion

This review aimed to provide the most recent and convincing evidence on the rehabilitation effects of e-Health interventions compared to conventional treatments for SLE patients. The results of this systematic review showed that e-Health interventions can improve health outcomes such as emotional status or/and quality of life in patients with SLE compared to control interventions such as usual care, SLE pamphlet, and other non-e-Health interventions. It is also important to highlight that this review considered the safety of patients with SLE who use e-Health tools in their interventions. None of the included studies reported adverse events after the interventions. Results suggest that e-Health interventions are safe and appropriate for patients with SLE.

Overall, 6 RCTs (743 participants) met the inclusion criteria. The structured e-Health interventions varied between the 6 studies included; therefore, the results of this systematic review are not specific to one type of intervention. Effectiveness was high across e-Health intervention types based on disease profile, satisfaction, and cost-effectiveness. It is relevant to report that there was variability in the type and design of the e-Health intervention. 

In the context of the COVID-19 pandemic, e-Health was adopted to provide care for patients with a diagnosis of chronic diseases, such as SLE. Results of this systematic review show a notable recent influx of published articles on e-Health delivery for the care of SLE patients since 2020, as the COVID-19 pandemic has significantly altered the clinical practices and management of chronic conditions such as SLE [13]. The successful use of e-Health in patients with SLE demonstrates the economic and social benefits of its use, as well as its clinical importance in the monitoring of these patients [27]. The findings of this study indicate that e-Health can effectively alleviate symptoms in patients with SLE. Additionally, there are many other potential advantages to using these types of tools to address SLE. Some of these include increasing the efficiency of the health workforce, leading to greater adherence to treatment, and the ability to provide people with a variety of higher quality health resources (e.g., knowledge, education) about SLE and personalized rehabilitation recommendations. An even more important aspect to highlight is that e-Health can overcome the geographical, economic, and time-related barriers that rehabilitation services sometimes pose, providing medical care to people from rural communities and patients with SLE who cannot attend traditional rehabilitation programs in person, which was considered essential during the COVID-19 pandemic [15].

In this systematic review, an e-Health intervention was considered to be a rehabilitation service using various telecommunication technologies. Currently, thanks to mobile phones, applications, virtual reality devices, and other electronic devices, it is possible to integrate e-Health technology into rehabilitation services in clinical practice.

An important consideration is that most participants in the studies had minimal disease activity (SLEDAI score less than 4) at baseline. Therefore, the overall results are not applicable to all people with SLE. Considering that people with SLE can experience varying symptoms and degrees of symptoms over time, the change in outcomes from baseline to the end of intervention needs to be read with caution (i.e., the change in outcome reporting might be a reflection of how the patient was feeling on the day of testing rather than a change in feelings before and after the intervention).

When analyzing the results of our systematic review, it is important to keep in mind the unique challenges faced by older populations and/or those with coexisting chronic diseases. As patients get older, they are at an increased risk of multiple chronic diseases, such as cardiovascular diseases, diabetes, and arthritis. This not only complicates the management of SLE but also predisposes patients to require comprehensive rehabilitation services [28,29]. Despite the broad scope of our review, most of the studies included predominantly featured younger participants, limiting the generalizability of our findings to older adults with SLE. This highlights the relevant gap that exists in the current research landscape, emphasizing the need for future studies. These need to specifically address the efficacy and adaptability of e-Health interventions among older SLE patients, who may benefit greatly from tailored e-Health solutions designed to manage both SLE and their additional chronic conditions.

Despite its strengths, our systematic review has a few limitations that should be mentioned. First, a literature search was conducted in the major electronic databases, but no other databases were searched. Therefore, additional relevant studies may have been missed. Second, due to the different scores in the methodological quality of the RCTs included, the reliability and validity of the overall results may have been affected. Furthermore, as a consequence of studies with methodological flaws, insufficient blinding, or small sample sizes, biases may arise that affect the precision of estimated treatment effects. Third, the number of relevant RCTs was limited, so it was not possible to perform a meta-analysis to estimate the overall effect. In addition, the heterogeneity among the different interventions limits the generality of the results.

### Clinical Implications

According to our results, e-Health intervention is an effective treatment for SLE patients. This treatment modality can enable SLE patients to manage their symptoms at any time and place in a timely and appropriate manner. In addition, it can provide accessible and continuous multidisciplinary therapeutic approaches for those who are unable to attend traditional face-to-face services. 

Studies of higher methodological quality and longer follow-up are needed to determine the efficacy of different types of e-Health interventions and to assess long-term outcomes in SLE patients. 

## 5. Conclusions

For patients with SLE, e-Health intervention is a safety approach to improve health outcomes. However, more high-quality studies with large samples are needed, with a focus on the long-term outcomes of e-Health intervention for patients with SLE. 

This systematic review will contribute to the literature by providing evidence of the benefits of e-Health intervention for the health outcomes of patients with SLE. It is of great relevance for clinical practice to provide interventions through e-Health to patients with chronic diseases such as SLE. E-Health should be used as a strategy to mitigate the economic costs, travel, and delays of usual interventions, and to obtain an immediate response to any question that SLE patients may have during their intervention process.

## Figures and Tables

**Table 1:** Characteristics of the studies included.

First Author (Country) and Year	Design	Aim	Participants (Women), Age (Mean ± Standard Deviation)	Duration of Disease in Years (Standard Deviation)	Intervention		Follow-Up (Months)	Instrument	Key Findings
					Experimental group	Control group			
Miedany et al. (USA) 2017 [25]	RCT (crossover)	Show the value of e-PROMs in the assessment and management of SLE over a 24-month period and its association with adherence to therapy, as well as organ damage, adjusted for potential confounding factors.	n: 147 (133), NR EG: 74 (67), 28.2 ± 8.3 CG: 73 (66), 29.1 ± 8.7	1.28 (0.3)	Online e-PROM questionnaire	Treatment as usual	12 and 24	SLEDAI; SLICC/ACR DI	E-PROMs vs. treatment as usual (NSD)
Singh et al. (USA) 2019 [22]	RCT	Tested the efficacy of the Individualized Decision aid for Diverse Women with Lupus Nephritis (IDEA-WON).	n: 298 (298), 37.3 ± 0.7 EG: 151 (152), 37.1 ± 1.0 CG: 147 (147), 37.6 ± 1.0	NR	Online decision aid	Lupus pamphlet	3	Decisional Conflict Scale; IPC-SF and APPC; Control Preferences Scale	Online decision aid > Lupus pamphlet (NSD)
Onengiya et al. (USA) 2020 [24]	RCT	Examine the feasibility and acceptability of the use of automated digital reminders and personalized prescribed treatment plans to improve medication adherence in young adults with cSLE.	n: 19 (16), 20.5 ± 1.6 EG: 8 (5), 20.5 ± 1.7 CG: 11 (11), 20.5 ± 1.6	4.8 (3.2)	Personalized prescribed treatment plan and automated digital reminders	Treatment as usual	1	MASRI	Digital reminders vs. treatment as usual (NSD)
Khan et al. (USA) 2020 [26]	RCT (pilot)	Demonstrate that the use of a mobile app for self-tracking of dietary, environmental, and lifestyle factors combined with telehealth coaching, results in enhanced quality of life for patients with SLE compared to standard care alone.	n: 34 (32), NR EG: 16 (15), NR CG: 18 (17), NR	NR	Mobile app, telehealth coaching and usual care	Treatment as usual	4	FACIT-F; BPI-SF; LupusQoL	Digital intervention > treatment as usual (*p* ≤ 0.005)
Ho et al. (China) 2022 [21]	RCT	Evaluate the short-term patient satisfaction, compliance, disease control and infection risk of TM for patients with LN during the COVID-19 pandemic.	n: 122 (111), 44.4 ± 11.5 EG: 60 (55), 44.1 ± 11.7 CG: 62 (56), 44.7 ± 11.5	15.1 (9.0)	Telemedicine follow-up	Treatment as usual	6	SLEDAI-2k and PGA; SLICC/ACR	TM > treatment as usual (*p* = 0.042)
Pasyar et al. (Iran) 2023 [23]	RCT	Evaluate the effects of supportive counseling via a smartphone on health anxiety and acceptance of disability in patients with SLE.	n: 123 (116), NR EG: 62 (56), 44.19 ± 9.69 CG: 62 (60), 48 ± 8.92	NR	Smartphone supportive counseling and routine nursing care and training	Treatment as usual	2	Health Anxiety inventory; Linkowski’s Acceptance Disability Scale	Smartphone supportive vs. treatment as usual (NSD)

Notes: AOD: Acceptance of Disability Scale; APPC: Active Patient Participation Coding Scheme; BPI-SF: Brief Pain Inventory-Short Form; CG: control group; cSLE: children with systemic lupus erythematosus; EG: experimental group; e-PROMs: electronic patient-reported outcome measures; FACIT-F: Functional Assessment of Chronic Illness Therapy-Fatigue; FU: follow-up; HA: health anxiety; IPC-SF: Interpersonal Processes of Care-Short Form; LLDAS: Lupus Low Disease Activity State; LN: lupus nephritis; LupusQoL: lupus quality of life; MASRI: Medication Adherence Self-Report Inventory; n: sample; NR: not reported; NSD: no significant differences; PGA: physician global assessment; RCT: randomized controlled trial; RPCT: randomized pilot controlled trial; SLE: systemic lupus erythematosus; SLEDAI-2K: Systemic Lupus Erythematosus Disease Activity Index 2000; SLICC/ACR DI: Systemic Lupus Collaborating Clinics Damage Index; TM: telemedicine. Methodological quality and risk-of-bias.

**Table 2:** Summary of the PEDro scale.

First Author (Country) and Year	P1	P2	P3	P4	P5	P6	P7	P8	P9	P10	P11	Total Score
Miedany et al. (USA) 2017 [25]	1	1	0	0	0	0	0	1	1	1	1	6
Singh et al. (USA) 2019 [22]	1	1	0	1	0	0	0	1	1	1	1	7
Onengiya et al. (USA) 2020 [24]	1	1	0	1	0	0	0	1	1	1	1	7
Khan et al. (USA) 2020 [26]	1	1	0	1	0	0	0	1	1	1	1	7
Ho et al. (China) 2022 [21]	1	1	0	1	0	0	0	1	1	1	1	8
Pasyar et al. (Iran) 2023 [23]	1	1	0	1	0	0	0	1	0	0	0	4

## Data Availability

Data are contained within the article or Supplementary Materials. The original contributions presented in the study are included in the article/Supplementary Materials, and further inquiries can be directed to the corresponding author/s.

## Supplementary Materials

The following supporting information can be downloaded at: https://www.mdpi.com/article/10.3390/healthcare12161603/s1, Supplementary Materials S1: Search strategy.
