# Supplementary material for: E-Health Interventions to Improve Health Outcomes in Patients with Systemic Lupus Erythematosus: A Systematic Review
Source: Healthcare (Basel). 2024 Aug 12;12(16):1603. doi: 10.3390/healthcare12161603 (PMC11353259; doi:10.3390/healthcare12161603)
Supplement: Supplementary file 1 [file healthcare-12-01603-s001.zip › healthcare-3053360-revised supplementary.pdf]

Supplementary Materials

Supplementary Materials S1. Search strategy.

MEDLINE via Pubmed:

("Systemic Lupus Erythematosus"[MeSH Terms] OR "Lupus Erythematosus, Systemic"[MeSH Terms] OR "Lupus Vasculitis, Central Nervous System"[MeSH Terms] OR "Lupus Erythematosus, Discoid"[MeSH Terms] OR "Lupus Coagulation Inhibitor"[MeSH Terms] OR "Lupus Nephritis"[MeSH Terms] OR "Lupus Erythematosus, Cutaneous"[MeSH Terms] OR "Panniculitis, Lupus Erythematosus"[MeSH Terms])) OR "Lupus Vulgaris"[MeSH Terms]) AND ("Virtual Reality Exposure Therapy"[MeSH Terms] OR "Virtual Reality"[MeSH Terms] OR "Libraries, Digital"[MeSH Terms] OR "User-Computer Interface"[MeSH Terms] OR "Exergaming"[MeSH Terms] OR "Telerehabilitation"[MeSH Terms] OR "Telemedicine"[Majr] OR "Augmented Reality"[Mesh] OR "Haptic Interfaces"[Mesh] OR "Telepathology"[Mesh] OR "Technology"[Mesh] OR "Remote Sensing Technology"[Mesh] OR "Wireless Technology"[Mesh] OR "Biomedical Technology"[Mesh] OR "Educational Technology"[Mesh] OR "Technology Transfer"[Mesh] OR "Technology Assessment, Biomedical"[Mesh] OR ("Technology"[Mesh] OR "Wireless Technology"[Mesh] OR "Biomedical Technology"[Mesh] OR "Educational Technology"[Mesh] OR "Digital Technology"[Mesh] OR "Disruptive Technology"[Mesh] OR "Information Technology"[Mesh] OR "Medical Informatics"[Mesh] OR "Medical Laboratory Science"[Mesh] OR "Biomedical Enhancement"[Mesh] OR "Self-Help Devices"[Mesh] OR "Wearable Electronic Devices"[Mesh] OR "Smartphone"[Mesh] OR "Tablets"[Mesh] OR "Computers, Handheld"[Majr] OR "Cell Phone" [Mesh] OR "Mobile Applications"[Majr])

Web of Science:

("Systemic Lupus Erythematosus" OR "Lupus Erythematosus, Systemic" OR "Lupus Vasculitis, Central Nervous System" OR "Lupus Erythematosus, Discoid" OR "Lupus Coagulation Inhibitor" OR "Lupus Nephritis" OR "Lupus Erythematosus, Cutaneous" OR "Panniculitis, Lupus Erythematosus" OR "Lupus Vulgaris") AND ("Virtual Reality Exposure Therapy" OR "Virtual Reality" OR "Libraries, Digital" OR "User-Computer Interface" OR "Exergaming" OR "Telerehabilitation" OR "Telemedicine" OR "Augmented Reality" OR "Haptic Interfaces" OR "Telepathology" OR "Technology" OR "Remote Sensing Technology" OR "Wireless Technology" OR "Biomedical Technology" OR "Educational Technology" OR "Technology Transfer" OR "Technology Assessment, Biomedical" OR "Technology" OR "Wireless Technology" OR "Biomedical Technology" OR "Educational Technology" OR "Digital

Technology" OR "Disruptive Technology" OR "Information Technology" OR "Medical Informatics" OR "Medical Laboratory Science" OR "Biomedical Enhancement" OR "Self-Help Devices" OR "Wearable Electronic Devices" OR "Smartphone" OR "Tablets" OR "Computers, Handheld" OR "Cell Phone" OR "Mobile Applications")
